# Thermoelectric generator powered timepiece circuit for rechargeable battery operation

**DOI:** 10.1038/s41598-024-59260-8

**Published:** 2024-04-15

**Authors:** Sam Methuselah Penumala, A. Karmel, G. Kanimozhi, Jagriti Khanwalkar

**Affiliations:** 1 School of Computer Science and Engineering, Vellore Institute of Technology, Chennai, Tamilnadu 600127 India; 2 Centre for Smart Grid Technologies, School of Electrical Engineering, Vellore Institute of Technology, Chennai, Tamilnadu 600127 India; 3 Laser Electronics Division, Raja Ramanna Centre for Advanced Technology, Rau-Rangwasa Rd, Indore, Madhya Pradesh 452013 India

**Keywords:** Lithium polymer battery, Thermoelectric generator, Battery powered watch, Seebeck effect, Energy science and technology, Engineering

## Abstract

The advent of digital technology has revolutionized the way we keep track of time. Digital watches have become an essential part of our daily lives and provide us with accurate and reliable time measurement. However, battery reliability is a long-standing issue in the digital watch industry. Batteries require frequent replacement and are a major source of waste. To solve this problem, a digital watch that runs on a lithium-polymer battery that is recharged by a voltage generated by a thermoelectric generator (TEG) placed on the hand. The proposed model uses TEG1-19913 that generates power in the range of 11.5 W to 14.5 W with hot end basking at 250 °C and a cold end between 30 °C and 50 °C. The TEG voltage is used to charge the lithium polymer battery, eliminating the need for conventional charging methods. The watch is designed to be compact and lightweight, so it can be worn comfortably for extended periods of time. The TEG is integrated into the watch strap and ensures that it is constantly in contact with the skin. The lithium-polymer battery used in the watch is a high-performance rechargeable battery that has high energy density and long life. In summary, the proposed digital watch is an innovative ecological solution to the problems associated with traditional battery-powered watches. The compact and light design of the watch combined with the energy-efficient display makes it a convenient and efficient timekeeping device.

## Introduction

In recent centuries, humans have focused primarily on increasing energy production to support the development of industry, transportation, and the overall quality of life. However, as a result of the recent energy crisis, there has been a shift towards more efficient energy management. This shift has contributed to the growing interest in thermoelectric generators (TEGs)^1^, which enable the collection of wasted thermal energy, power generation in extreme environments, electrical power generation in remote areas, and microfabrication for sensors. TEGs have the potential to directly convert waste heat energy into electrical energy, making them a promising green technology. By using TEGs to convert waste heat energy into electricity^1,2^, the overall efficiency of energy conversion systems can be improved. A significant amount of waste heat is produced by various industries, including energy companies and manufacturing plants. As a result, much of TEG research has focused on finding suitable thermoelectric materials that can withstand the high temperatures of industrial heat sources at a reasonable cost and with good performance. Further TEG research is still needed to achieve these goals.

TEGs work on the principle of the Seebeck effect^1^, that states when two different types of metals are joined together and one end is heated, a voltage is produced. This voltage is proportional to the temperature difference between the two ends of the metals. TEGs are composed of thermoelectric materials that have a strong thermoelectric effect, meaning they can generate large voltages in response to a temperature difference. These materials are typically semiconductors such as bismuth telluride or germanium silicon. Thermoelectric materials are sandwiched between two conductors, such as copper or aluminum, which act as the hot and cold junctions of the device.

**Figure 1:**
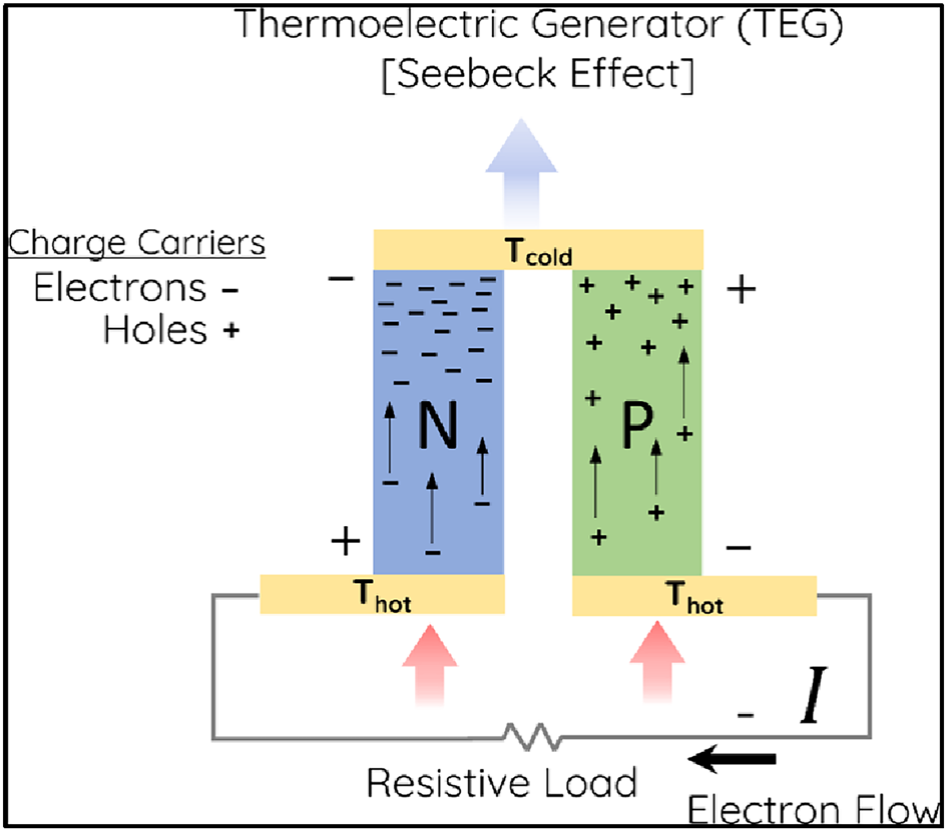
Working of TEG.

When a temperature difference is applied to the TEG, the thermoelectric materials generate a voltage, as seen in Fig. 1. This voltage can be used to power an electrical load. In order to generate electricity, a temperature difference must be maintained across the TEG. This can be achieved in a number of ways, such as using a heat source and a heat sink, or using the ambient temperature as the heat sink and an external heat source as the heat source.

The efficiency of a TEG depends on a number of factors, including the temperature difference between the hot and cold junctions, the thermoelectric material used, and the electrical load. TEGs are usually not very efficient, with efficiencies ranging from a few percent to around 10%. Thermoelectric watches are a relatively new type of watch that has gained popularity in recent years due to its unique way of producing energy and its ecological nature. This watch uses the thermoelectric effect that occurs when there is a temperature difference between two dissimilar materials, to generate electricity and power the watch. This allows them to operate without the need for a traditional battery or manual winding. In this work, the investigation is focused on the thermoelectric phenomenon and its application in thermoelectric watches. This article also dive into the potential pros and cons of using this technology in watches and compare it to traditional methods of power generation in watches. Since the energy crisis, academics and businessmen have focused on improving energy management, particularly energy system efficiency. This explains TEG's rising popularity. Today, TEGs can gather lost thermal energies, manufacture energy in severe conditions, generate remote electric power, and micro production for sensors. Thermoelectric power generators can directly transform waste-heat energy into electricity, making them a viable green technology. This alternative green technology may boost energy conversion system efficiency by transforming waste-heat energy into electricity. Power utilities and factories release a lot of waste heat. Thus, industrial waste heat use has dominated research. TEG research must identify thermoelectric materials that can resist increasing industrial heat source temperatures at a reasonable cost and operate well. 

## Literature survey

Thermoelectric watches^2^ are an emerging technology that aims to harness the heat generated by the human body to power electronic devices. These watches have the potential to revolutionize the way we think about energy generation and consumption, as they offer a sustainable and renewable energy source that is both environmentally friendly and convenient. In recent years, the development of thermoelectric materials and advancements in microelectronics have paved the way for the creation of small-scale energy-harvesting devices such as thermoelectric watches. This literature survey aims to provide an overview of the current state-of-the-art in the field of thermoelectric watches. The survey will cover the latest research and development in thermoelectric materials, energy conversion efficiency, and temperature monitoring capabilities. The survey will also examine the challenges and limitations associated with the design and development of thermoelectric watches, as well as the potential applications and future directions of this technology.

The goal of the research outlined in^3^ is to create and study a wristwatch that is fully powered by the heat generated by the human body. This type of device has never been produced before and represents a unique and innovative approach to powering electronic devices. Research shows that the maximum output of the generator is 2 mW, which is achieved at a temperature difference of more than 5 °C. The maximum output power per square centimeter is 0.22 mW. Although this level of power output is relatively low, it is still an important step forward in the development of energy sources powered by body heat. However, the research also notes that the efficiency of this type of device is relatively low and may not be effective in a hot climate like India. This highlights the need for further research and development to improve the efficiency and effectiveness of body heat powered energy sources. In^4^, a commercial F-TEG (flexible TEG) was worn on the lower leg to collect body thermal energy while the user performed various physical activities. The results of this study showed that it was possible to trace the semi-stationary parts of the generated signal back to the activity being performed. This suggests that it may be possible to use energy sources powered by body heat to power electronic devices in a more targeted and specific way depending on the activity being performed. F-TEGs are more efficient than conventional TEGs, but are difficult to obtain and require further research. This suggests that there is still much to learn about the potential and limitations of energy sources powered by body heat.

In^5^, the performance of obtaining human waste heat using TEGs was investigated. The study analyzed three commercially available low-voltage boost converters in combination with a series of five thermoelectric elements placed on the bottom of the leg. The results showed that a constant 443μW and 175mJ were generated during a 6-minute walk indoors. This shows that it is practical to store up to 6% of the generator's maximum power for real-world use. Overall, the research discussed in these three studies suggests that body heat-powered energy sources have the potential to be a viable and sustainable option for powering electronic devices. However, further research is needed to fully understand their potential and limitations and to improve their effectiveness and efficiency.

One of the main problems with thermoelectric coolers (TECs) is their limited coefficient of performance (COP) and thermal output. However, these limitations can be overcome by using a multi-stage thermoelectric cooler. In^6^, the authors performed a study of heat transfer through a TEC using a multi-stage thermoelectric module. Using a multi-stage thermoelectric chiller/generator can increase the COP. Jurkans et al.^7^ presents a model that is calculated by numerically solving the governing equations of heat transfer using ANSYS thermoelectric simulation. The results of this research can be used to optimize the structural design of wearable TEGs and to select materials that improve energy production for mobile devices powered^8^ by body heat. Shi et al.^9^ presents a novel wearable TEG with a copper foam heatsink to power the electronics required for body motion detection. The TEG with a copper foam heat sink had the highest power-to-weight ratio of 30.73 μ Wg^-1^ at a temperature difference of 45 K. This TEG would be suitable for body-worn and heat-harvesting applications. In^10^, the authors developed a design prototype to investigate human energy obtained from different parts of the body. The research showed that for human temperatures of 36 °C or higher, the prototype was able to generate an output voltage of more than 2.8 V, confirming that the energy from human heat can be used to generate electricity. Wang et al.^11^ describes two collaborative TEC-TEG cooling systems, namely the PCC system and the VCC system, which provide an alternative approach to improve the performance of TEC-based cooling systems. These systems show superior performance compared to Peltier cooling systems. Selvan et al.^12^ proposes a microfabrication technique for Cu-Co planar, lateral, and thick-film TEGs. A sandwich planar structure can harvest higher voltage (Voc) and power (Pmax) at lower temperature differences (ΔT) and resistance (RE) by stacking more thermocouples at the same length and width. However, more research needs to be done on this technology before it can be used for practical energy applications. In^13^, the authors modeled a TEG integrated with a wireless handset and numerically investigated its performance. With a matched load of 1.1 ohms, the TEG provided 0.6 mW of electrical power at a temperature difference of 10.5 K, with hot and cold side temperatures of 305.15 K and 294.65 K. Although an actual prototype was not developed, this work provides insight into how to simulate and work with TEG for real applications.

In^13^, the authors propose a unit called CTEG-PCM, which is designed to solve the self-powering problem of wireless sensor network nodes in forests. The unit is based on the use of a TEG and a phase change material (PCM) to store and release energy as needed. The authors conducted experimental tests of the CTEG-PCM under open circuit conditions and found it to be effective in generating power for wireless sensor nodes in a forest environment. In^14^, the authors decided to analyze the valley-spin thermoelectric properties and the Nernst coefficient at two different temperatures for a ferromagnetic silicene superlattice. The results of the study showed oscillatory behavior for all investigated parameters, suggesting that ferromagnetic silicene superlattices^15^ could be a promising material for flexible thermoelectric power generation. In^16^, the authors chose a different approach, hybridizing graphene with metal nanoparticles (Au, Ag and Pt). The resulting hybridized graphene showed a significant decrease in sheet resistance and a 25% decrease in Seebeck coefficient, but a threefold increase in thermoelectric power factor due to the reduction in sheet resistance. Wu et al.^17^ presents an embedded MPPT boost converter using a FOCV scheme focused on a TEG energy harvesting system. The converter is designed to be highly efficient, with a peak end-to-end efficiency of 72.1% at an open-circuit voltage of 170 mV and an input power of 34.4 µW. The ultra-low supply voltage and TD-based control enable the system to have low power consumption and high efficiency performance, making it a useful option for powering electronic devices using TEGs. Overall, the research described in these four studies offers a number of approaches and solutions for harnessing and generating energy from thermoelectric materials. From the use of PCMs for energy storage and release to research into new materials and hybridization techniques, these studies demonstrate the potential of thermoelectric power generation as a sustainable and efficient power source for electronic devices.

Yan et al.^18^ suggested a MEMS-based thermoelectric and photoelectric integrated power generator. To optimize the heat flow, the square arrays of thermocouples are thermally insulated on both sides with an oxide passivation layer on the lower side and a polyimide thermal insulation layer on the upper side of the thermocouple. The highest output voltage factor and power factor of the -TEG are 0.149 V cm^−2^ K^−1^ and 3.03 × 10^−3^ µW cm^−2^ K^−2^, respectively, when the front side of the device is coated with a sputtered Al membrane. The measured photoelectric conversion efficiencies for the front and rear of the device are 4.45% and 0.682%, respectively. Several wireless sensor nodes, including temperature sensors and flow sensors, may be powered by the electrical energy that has been gathered.

## Proposed work

The use of mobile electronic gadgets, such as smartphones, watches, and even smart clothing, permeates every aspect of modern life. Unfortunately, the development of batteries as energy sources has not been as rapid. Consumers find devices that don't require routine charging or battery replacement to be more appealing and competitive. With a few minor exceptions, such autonomous gadgets are sadly uncommon. They are made possible by integrating rotor (kinetic) inductors for electronics that are sensitive to vibrations and solar cells for devices that are exposed to sunlight. Rarely is heat energy from the environment, industry, or human body utilised to provide electricity to different users. If the human body could do the same work as a watch battery with cleaner, more recyclable technology, that is a win for the environment. Thus, the goal is to create a wristwatch that runs only on body heat. The challenges faced are temperature difference and working with the small millivolts generated. India, being a tropical and hot country, the temperature difference between the ambient outer environment and the human body is less than 10˚C. This will lead to less generation of the voltage. This can be overcome using efficient TEGs that can work with such less ∆T. There are times when the environment temperatures will be higher than our body temperatures which will generate a negative voltage.

**Figure 2:**
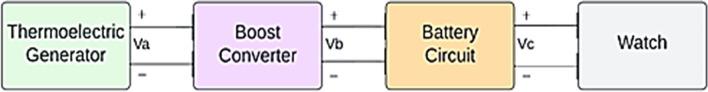
Block diagram of the proposed work.

**Figure 3:**
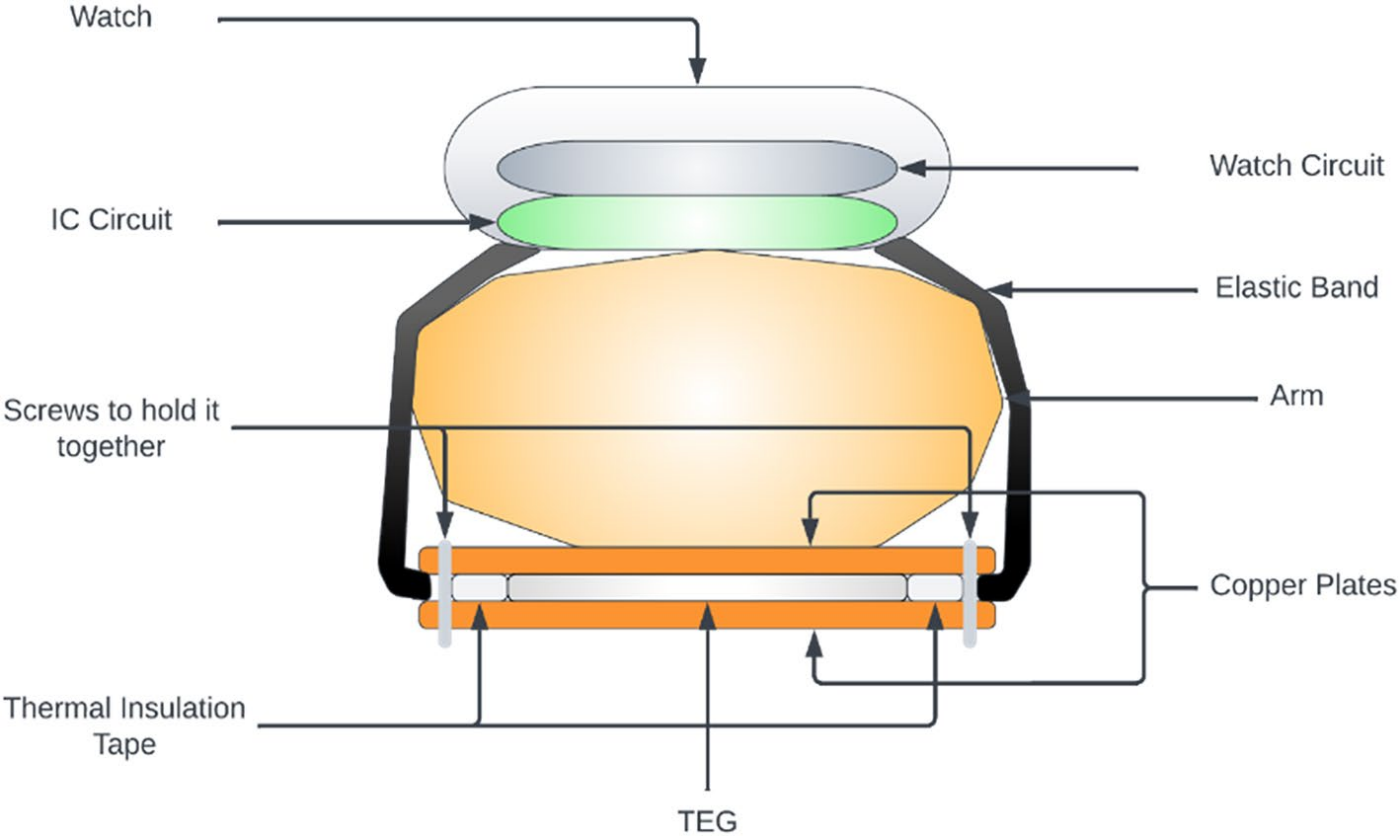
TEG—Prototype.

The IC should be capable of working at such small potential differences. It is recommended to work with at least 20% higher voltage than the minimum operating voltages. Many existing numerical models for TEGs performance prediction, design and optimization assume that the material properties are constant from the hot-end to the cold-end, and the variation of material properties with temperature along the TE element, electrical and thermal contact losses at the junctions are usually neglected. This can cause a lot of problems in real life situations. Furthermore, the research done in all the papers and articles tend to provide results when the temperature differences are very high between the cold side and hot side of the TEG. TEGs do not seem to stabilize and give a constant output until and unless given the ideal conditions to work with, which is not practical in real-life scenarios. Thermoelectricity has great potential for increasing energy efficiency by turning waste heat into electricity and, run backward, in solid-state refrigeration applications. It relies on the thermoelectric effect such that a temperature gradient across a material result in an electrical voltage. The physics behind it is that hot electrons are moving faster, so they tend to diffuse away from the hot end of the metal and toward the cool end. The main advantages of TEGs^19–22^ include being a dependable energy source, environmentally benign, highly scalable, which allows for application to heat sources of any scale, lowering manufacturing costs, and recycling unused heat energy. Figure 2 represents the block diagram of the proposed work and Fig. 3 show the complete design of TEG prototype developed.

## Materials

**Figure 4:**
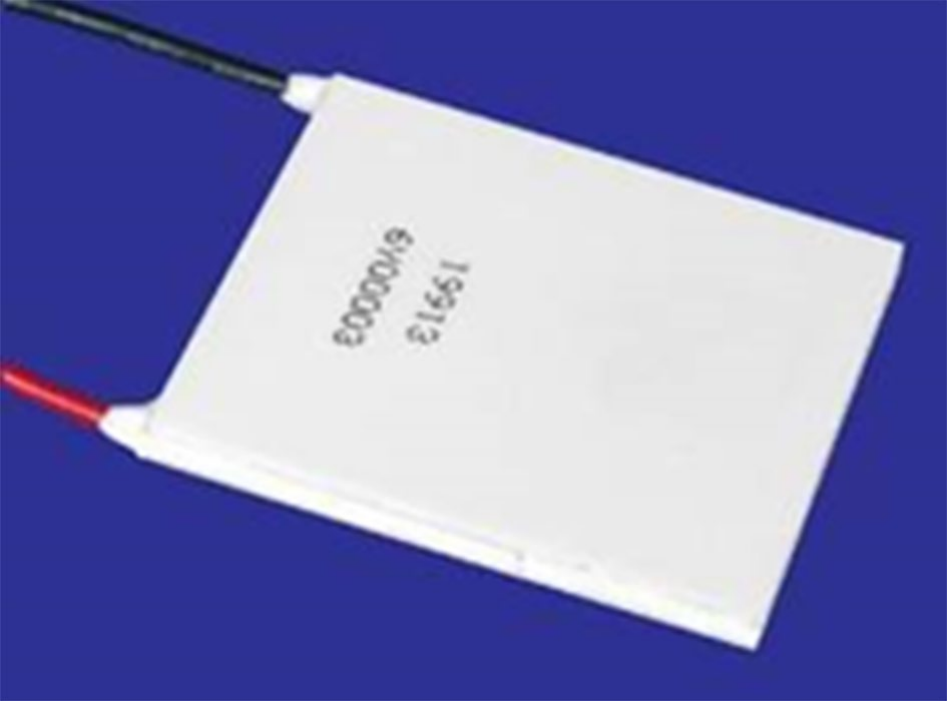
Proposed TEG1-19913 for timepiece circuit.

**Figure 5:**
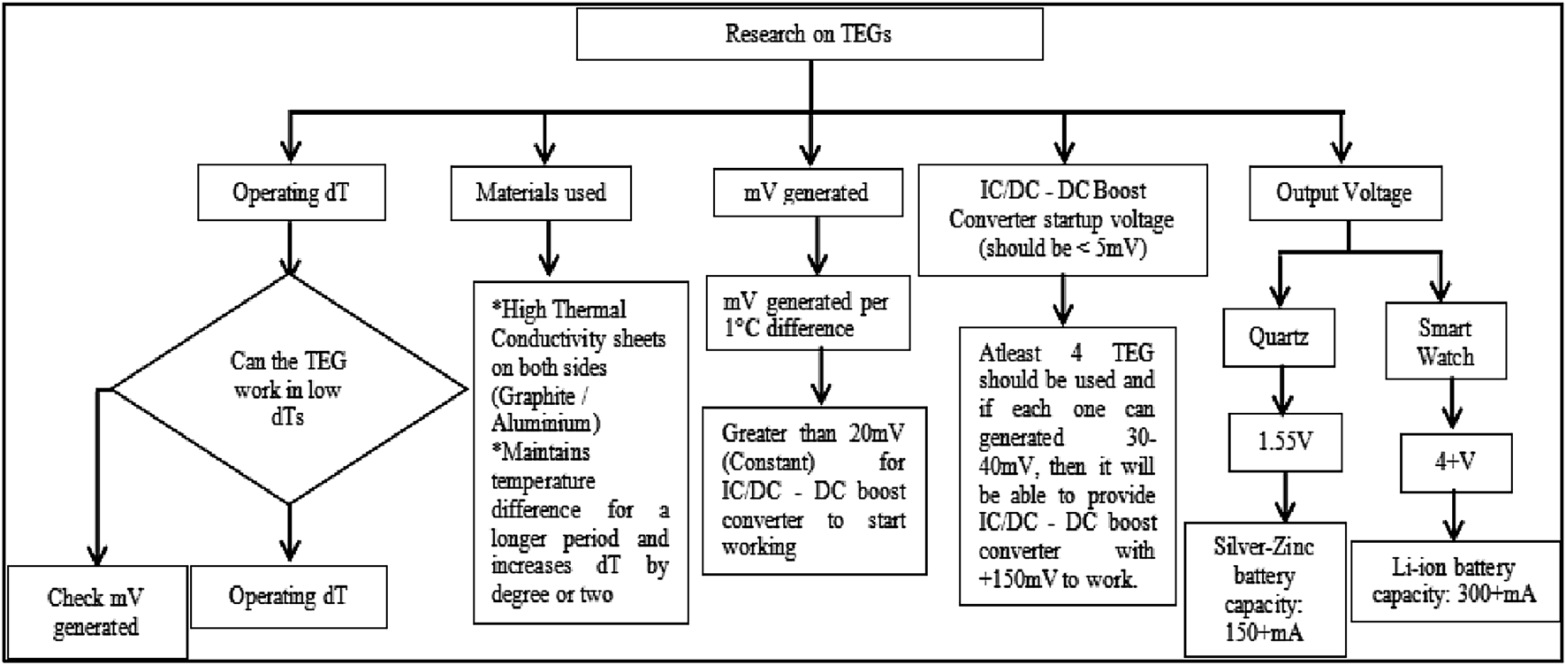
Flowchart of research on TEGs.

Thermoelectric Generator (TEG) is a device that converts temperature differences into electrical energy. Figure 3 show the complete design of TEG prototype developed. By placing the generator in a specific manner, with the hot side facing the body or arm and the cold side facing the environment, a temperature difference is created, which generates a potential difference. In this system, a custom TEG1-19913, with dimensions of 50 mm × 50 mm × 3.8 mm as seen in Fig. 4, is being used. This generator has 374 thermocouples, which is significantly more than any other generator of its size, enabling it to generate 60–70 mV for a 2 °C temperature difference.The invention at hand consists of four main components: TEG, step-up circuit (IC), the battery circuit, and watch circuit. Of these components, the step-up circuit is the most critical, as it is responsible for increasing the voltage generated by the TEG to a usable level. However, to achieve a good temperature difference and generate more voltage, it is important to address the issue of thermal leakage from the hot side to the cold side. Proper mounting is crucial to maintain a temperature difference between both sides. To achieve this, thermal insulation tape is used to isolate both sides of the generator. Figure 5 represents the research learnings on TEGs.

### Mathematical analysis of TEG1-19913 performance

This analysis provides a framework for mathematically modeling the TEG1-19913 performance.

The Seebeck effect relates the voltage generated by a TEG to the temperature difference between its hot and cold sides:

1 $${\text{V}} = \alpha *\Delta {\text{T}}$$

where V is the output voltage (V), α is the Seebeck coefficient (V/K), ΔT is the temperature difference between the hot and cold sides (K). The TEG's internal resistance affects its output power. It can be modeled as a combination of: AC internal resistance (R_a): Provided in the datasheet (1.58 Ω at 27 °C). Peltier resistance (R_p): Related to the Seebeck coefficient through the following equation,

2 $$R_{p} = 1/(\alpha^{2} *\sigma )$$

where σ is the electrical conductivity (S/m). The total internal resistance (R_t) at a specific temperature can be calculated as:

3 $${\text{R}}\_{\text{t}} = {\text{R}}\_{\text{a}} + {\text{R}}\_{\text{p}}$$

The maximum output power (P_max) can be estimated using the following equation:

4 $$P_{m} ax = V^{2} /(4*R_{t} )$$

where V and R_t are obtained from the previous steps.

To achieve maximum power transfer, the load resistance (R_L) should match the internal resistance of the TEG:

5 $${\text{R}}\_{\text{L}} = {\text{R}}\_{\text{t}}$$

The TEG's efficiency (η) relates the electrical power output to the thermal power input:

6 $$\eta = {\text{P}}\_{\text{out}}/{\text{Q}}\_{\text{in}}$$

where, P_out is the electrical power output (W), Q_in is the thermal power input (W). The datasheet provides a range for the thermoelectric conversion efficiency (5.0–5.7%).

Additionally, 2 mm copper plates are employed to increase the thermal conductivity on both sides. These plates are secured in place using insulated screws and mounted on a rubber belt to ensure proper contact with the body, due to the elasticity of the rubber. By implementing these measures, the TEG can efficiently convert the temperature difference into electrical energy.

**Figure 6:**
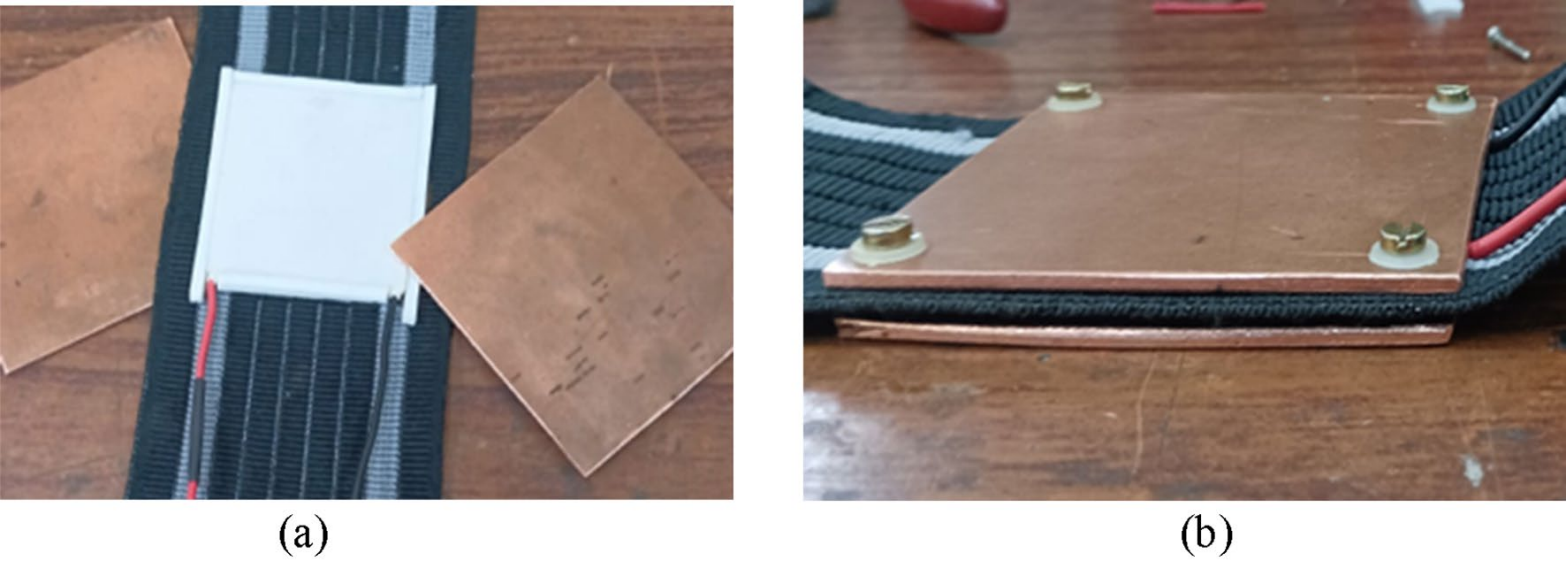
Mounting of TEG with copper plates on each side. (**a**) before fixing Cu plate, (**b**) after fixing Cu plate.

The use of high thermal conductivity plates on either side of a TEG can improve the efficiency of the device in several ways. Firstly, it can reduce the temperature gradient across the TEG, leading to a smaller thermal resistance and a more efficient transfer of heat. Secondly, it can help to evenly distribute heat across the TEG, ensuring that all the semiconductors are operating at the same temperature, which can reduce thermal stress and increase the device's reliability. Thirdly, high thermal conductivity plates can help to dissipate excess heat from the TEG, preventing the device from overheating and potentially damaging the semiconductors. In conclusion, the use of high thermal conductivity plates on either side of a TEG can improve its efficiency by reducing the thermal resistance between the hot and cold sides, ensuring even heat distribution, and preventing overheating. The choice of materials for the plates is crucial in achieving optimal performance, and careful consideration should be given to factors such as thermal conductivity, electrical insulation, and compatibility with the operating temperature range of the TEG. Figure 6a,b shows the mounting of TEG before and after fixing with copper plates on each side, respectively.

**Figure 7:**
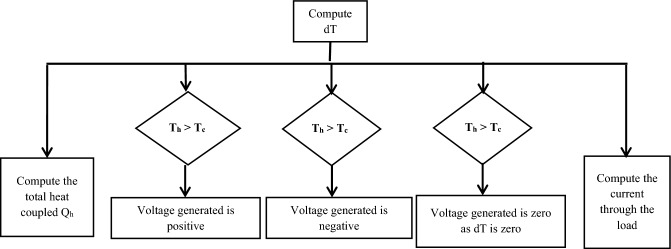
Flowchart to compute different temperature cases.

The flowchart (refer Fig. 7) gives the equations to calculate various variable for the three different cases possible while using a TEG^23,24^.

7 $$dT={T}_{h} - {T}_{c}$$

8 $${V}_{g}=S * dT$$

9 $${Q}_{h}=(S * {T}_{h} * I) - (0/5 * {I}_{2} * {R}_{C} ) - ({K}_{c} * dT )$$

10 $${C}_{l}=\frac{{R}_{C}}{{R}_{L}}$$

where ${T}_{h}$—Temperature on hot side, ${T}_{c}$—Temperature on cold side, $S$—the average seeback coefficient in volts/kelvin, $dT$—the difference in temperature across the couple in K, ${V}_{g}$—Voltage generated, ${Q}_{h}$—the input heat in Watts, ${K}_{c}$—the thermal conductance of the couple in Watts / K,${C}_{l}$—the generator output current in Ampere, ${R}_{C}$—the average internal resistance of the thermoelectric couple in Ohms, ${R}_{L}$—the load resistance in Ohms.

### Step-up circuit

**Figure 8:**
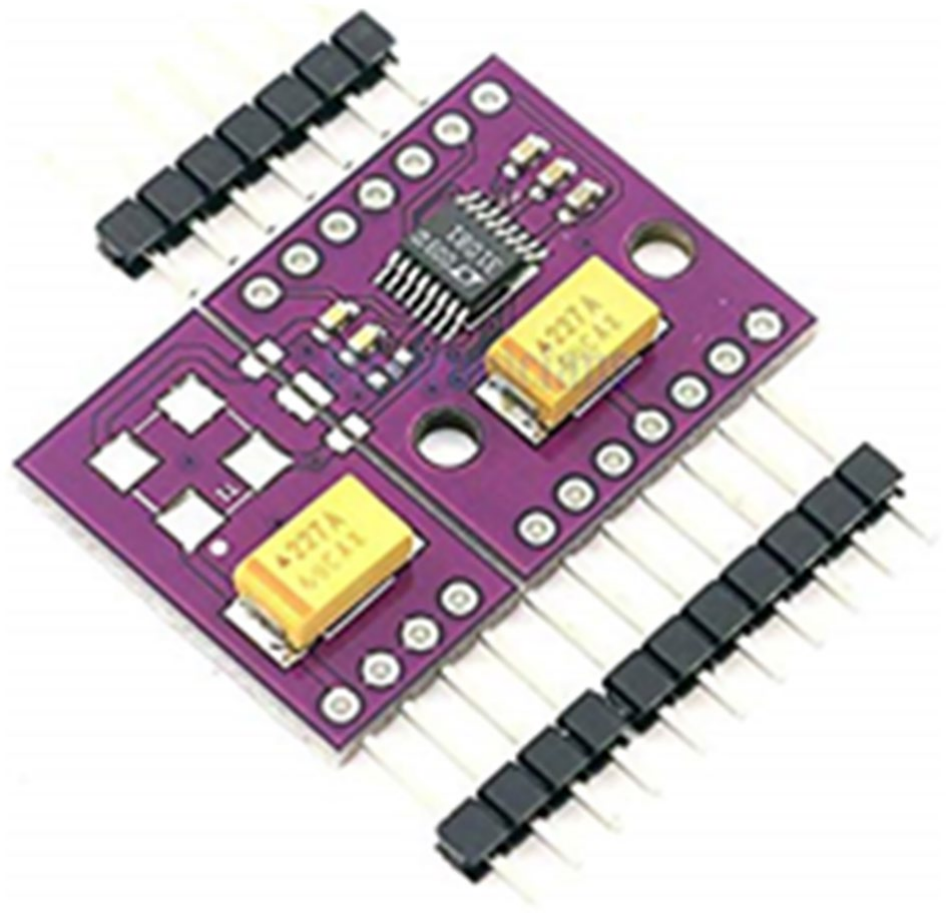
LTC3108 Breakout Board.

**Figure 9:**
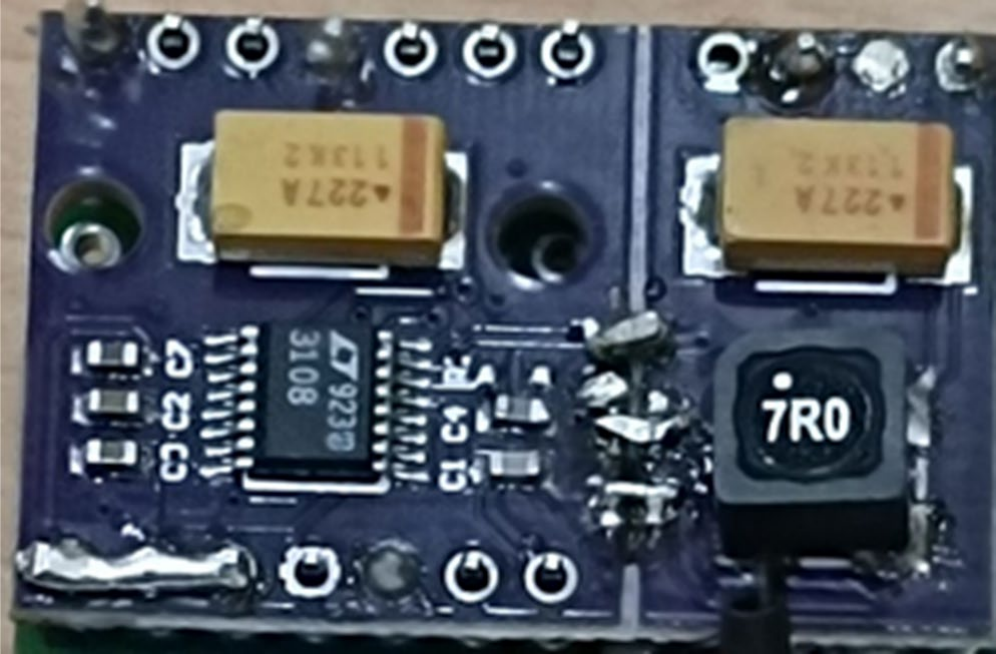
1:100 Transformer mounted on the Breakout Board.

The step-up circuit utilized involves the use of the LTC3108 (shown in Fig. 8), a highly integrated DC/DC converter that is particularly suited for harvesting and managing surplus energy from extremely low input voltage sources such as the TEG being used. The circuit employed involves the use of a breakout board for the IC, that includes the necessary capacitors as seen in Fig. 9. The LTC3108 offers the option to utilize 1:20, 1:50, and 1:100 transformers.

To obtain the maximum output possible, a 1:100 transformer (74,488,540,070) is utilized, which results in the TEG's output being stepped up by a factor of 100 as seen in Fig. 6. The next step involves programming the output of the IC. The LTC3108 provides four programmable outputs, including 2.35 V, 3.3 V, 4.1 V, and 5.0 V. As the output will be connected to a rechargeable battery with a rating of 3.7 V, the available options are limited to 4.1 V and 5 V. Since the current generated is minimal, it would take several hours to charge the battery. Therefore, the maximum output of 5 V is chosen, as you can see the first three points are shorted in the bottom left in Fig. 6, to charge the battery as quickly and efficiently as possible. The efficacy of a converter's start-up is heavily influenced by the turn ratio of its step-up transformer, which determines the minimum input voltage required for activation. By employing a 1:100 turn ratio, for instance, start-up voltages as low as 20 mV can be achieved. Additional factors affecting performance are the DC resistance of transformer windings and the inductance of the windings. The input capacitance at pin C2, typically 30pF, in parallel with the transformer secondary winding shunt capacitance. The recommended resonant frequency is in the range of 10 to 100 kHz.

### IC- LTC3108

**Figure 10:**
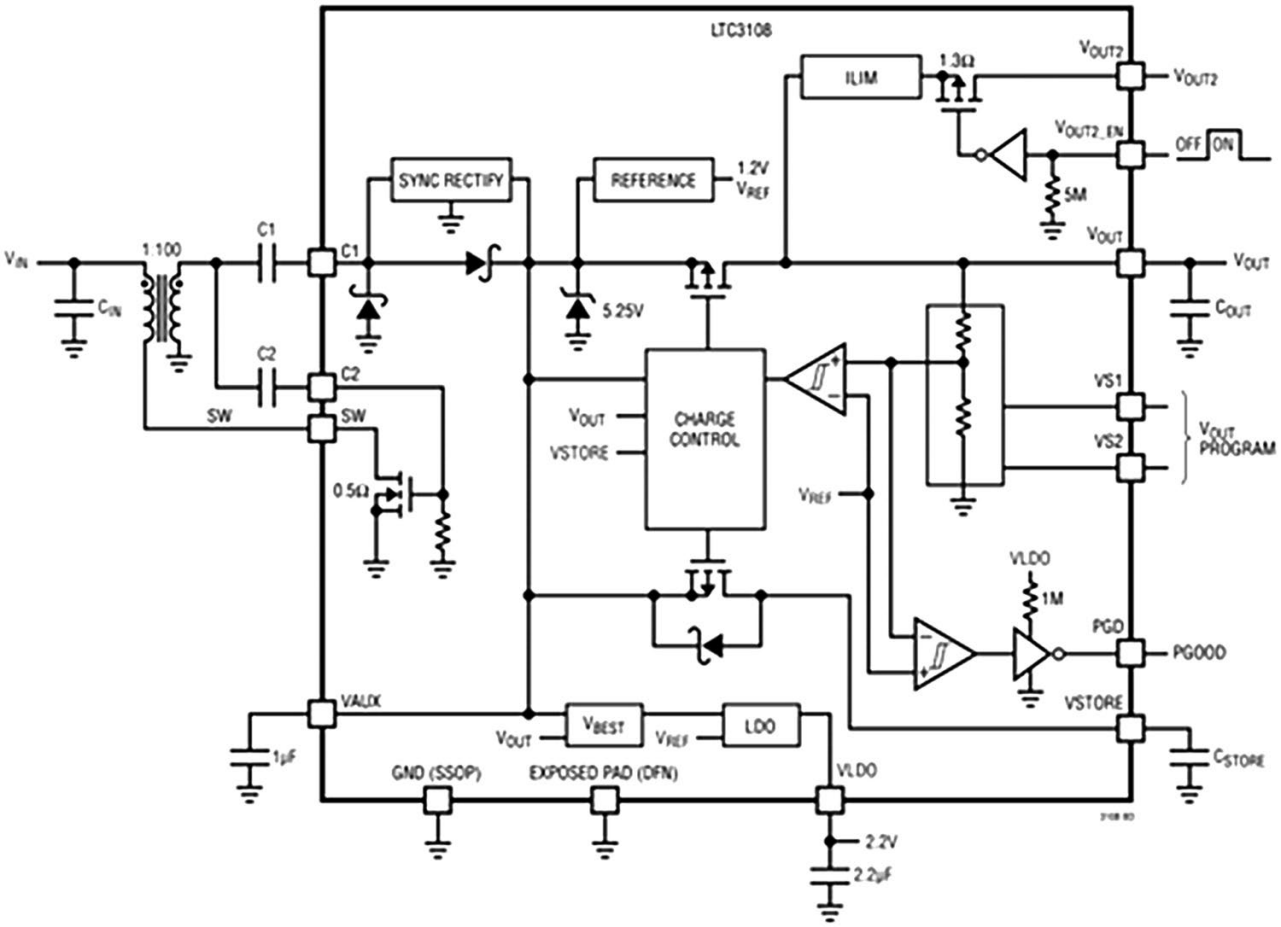
Block Diagram of LTC3108.

The LTC3108 portrayed in Fig. 10 operates on a boost converter topology, which consists of an inductor, a diode, and a capacitor. When the input voltage is applied to the inductor, a magnetic field is generated that stores energy. When the diode is switched on, the energy stored in the inductor is transferred to the output capacitor, resulting in a higher output voltage. The LTC3108 also features a low dropout regulator (LDO) that regulates the output voltage to a fixed voltage level. The LDO ensures that the output voltage remains stable even when the input voltage fluctuates. This is particularly important for low-power applications where a stable output voltage is necessary for the proper functioning of the electronic device. These include a power good output, an enable input, and a low quiescent current of less than 500nA. The power good output signals when the output voltage has reached a stable level, while the enable input allows the LTC3108 to be turned on and off as needed. The low quiescent current ensures that the LTC3108 does not consume too much power when it is idle.

The LTC3108 has a wide input voltage range of 20 to 400 mV and can produce output voltages of up to 5 V. It has an efficiency of up to 85%, which is one of the highest in its class. The LTC3108 also has a low shutdown current of less than 10nA that ensures that it does not consume power when it is not in use. To provide holdup when the input power in case of failure, the VSTORE capacitor have a very high value (thousands of microfarads or even Farads). The holdup capacitor has a functioning voltage rating of at least 5.5 V at the temperature at which it can be used since it can charge up to 5.25 V (independent of the settings for VOUT).

### Battery circuit

**Figure 11:**
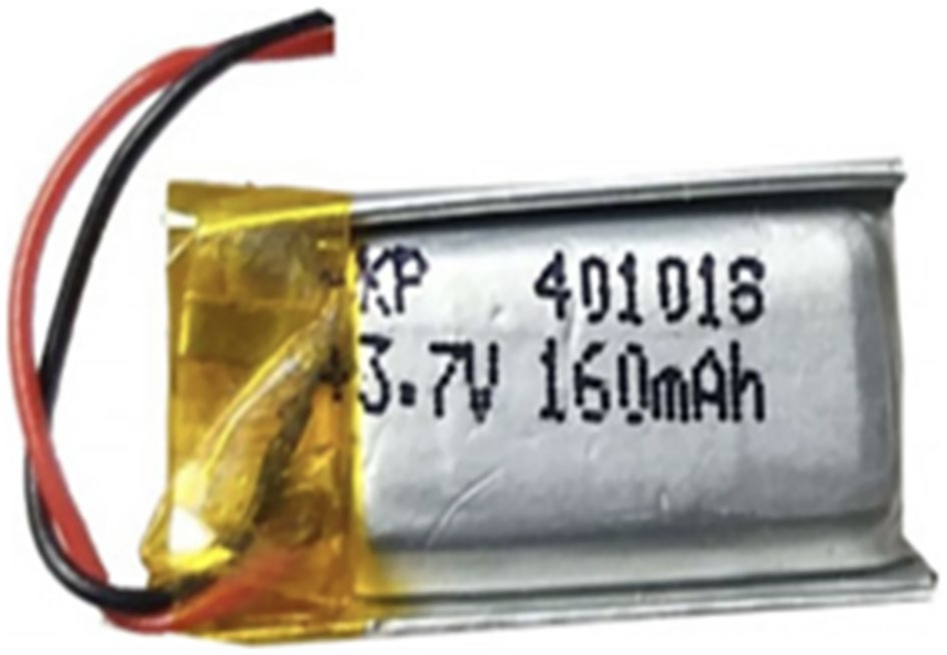
Lithium Polymer Battery.

**Figure 12:**
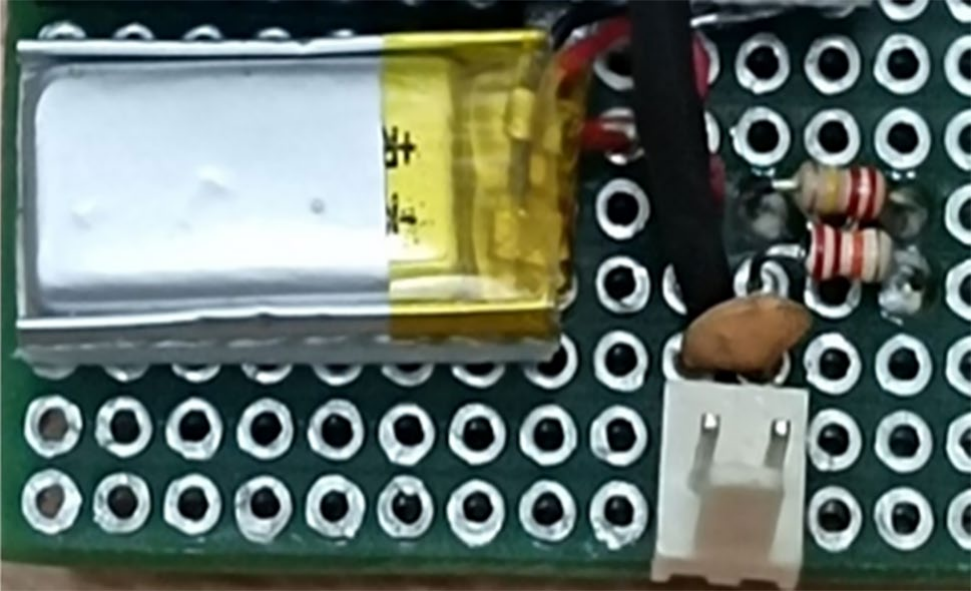
Lithium Polymerbattery circuit.

Lithium polymer (LiPo) batteries (shown in Fig. 11) are rechargeable batteries that have gained popularity in recent years due to their high energy density, lightweight, and flexibility in terms of shape and size. They are commonly used in portable electronic devices, such as smartphones, tablets, and laptops, as well as in drones, electric vehicles, and even medical equipment. The basic structure of a LiPo battery (shown in Fig. 12) consists of a positive electrode, a negative electrode, and an electrolyte. The positive electrode is typically made of lithium cobalt oxide (LiCoO_2_), while the negative electrode is made of graphite. The electrolyte is a polymer gel that acts as a separator between the two electrodes, allowing the flow of ions between them during charging and discharging cycles. One of the key advantages of LiPo batteries is their high energy density, which is the amount of energy that can be stored per unit volume or weight. This allows them to deliver more power than other types of batteries, making them ideal for use in high-performance devices. Additionally, they have a low self-discharge rate, which means that they can hold their charge for longer periods of time than other rechargeable batteries.

Another advantage of LiPo batteries is their flexibility in terms of shape and size. Unlike traditional cylindrical or rectangular batteries, LiPo batteries can be made in a variety of shapes and sizes, allowing them to fit into devices with unique shapes and sizes. This also allows for greater design flexibility, as device manufacturers can optimize the battery shape to fit the specific requirements of their product. However, there are also some disadvantages to LiPo batteries. One of the main concerns is their safety, as they can be prone to overheating and catching fire if not handled properly. This is due to the flammable electrolyte used in LiPo batteries, which can ignite if the battery is damaged or overcharged.

To mitigate this risk, LiPo batteries are typically equipped with safety features, such as thermal fuses and overcharge protection circuits. In conclusion, LiPo batteries are a popular choice for portable electronic devices and other applications that require high power density and design flexibility. While there are some safety concerns associated with LiPo batteries, proper handling and safety features can minimize these risks. Overall, the benefits of LiPo batteries make them an attractive choice for many different applications. A rechargeable LiPo battery in Fig. 8 with a rating of 3.7 V is utilized to power the watch circuit. The output of the LTC3108 IC is connected to the battery terminals, providing a charging mechanism, and simultaneously the terminals serve as the power source for the watch circuit.

It should be noted that the watch circuit requires a voltage of 3.015 V to operate efficiently. Therefore, to reduce the voltage provided by the battery to this level, 240kΩ resistors are used in series. While the resistors do indeed drop the voltage, the output obtained is not entirely stable. To overcome this issue, a 0.1 µF capacitor is placed in the circuit to ensure a stable output as can be seen in Fig. 9. This arrangement ensures that the watch circuit operates with the correct voltage, thereby prolonging the life of the circuit and improving overall efficiency.

### Thermal insulation tape around the TEG

Thermal insulation tape is a material that has a low thermal conductivity, which means that it is a poor conductor of heat. By wrapping the TEG with thermal insulation tape, the tape acts as a barrier to reduce heat transfer between the hot side and the surrounding environment. This results in a reduction of heat loss from the hot side and an increase in the temperature difference between the hot and cold sides of the TEG, leading to a higher electrical power output. The reduction of heat loss from the hot side to the ambient environment can be further explained by the concept of thermal resistance. In a TEG, the thermal resistance between the hot side and the ambient environment can be reduced by increasing the thickness of the insulation layer or by using a material with a lower thermal conductivity. This reduces the amount of heat that is lost to the ambient environment and increases the amount of heat that is available to generate electricity. In addition to reducing heat loss, using thermal insulation tape around a TEG can also protect the device from external factors that can negatively impact its performance. For instance, thermal insulation tape can prevent the ingress of moisture, dust, and other contaminants, that can degrade the performance of the TEG. It can also protect the device from mechanical damage and vibration, which can cause mechanical stress and reduce the device's reliability. It is worth noting that the choice of thermal insulation tape is important in achieving optimal performance. The tape should have a low thermal conductivity, be resistant to high temperatures, and be compatible with the operating temperature range of the TEG. In addition, the tape should be able to withstand the environmental conditions of the application, such as exposure to moisture, chemicals, and UV radiation.

In conclusion, the use of thermal insulation tape around a TEG can increase its efficiency by reducing heat loss from the hot side to the ambient environment. This results in a higher temperature difference between the hot and cold sides of the TEG, leading to a higher electrical power output. The choice of thermal insulation tape is crucial in achieving optimal performance, and careful consideration should be given to factors such as thermal conductivity, temperature resistance, and environmental compatibility.

### Mylar sheet in TEG

Mylar is a brand name for a type of polyester film that is widely used in a variety of applications, including electronics, packaging, and imaging. Mylar sheets are thin, flexible, and transparent, and they have excellent mechanical, electrical, and thermal properties. As a result, they are often used as a dielectric material in capacitors and other electronic components, as well as for insulation and protection of various components in a circuit. In electronic circuits, mylar sheets are used to provide insulation for various components, such as capacitors, transformers, and coils. These components can generate heat during operation, which can lead to thermal stress and degradation of performance over time. In addition, these components can also generate electromagnetic interference (EMI), which can interfere with the operation of other components in the circuit. Mylar sheets can help to address these issues by providing a barrier that separates the components from each other and from the external environment.

Mylar sheets are also resistant to moisture, chemicals, and UV radiation, which makes them ideal for use in harsh environments. They are non-reactive and non-conductive, which means that they do not interact with other components in the circuit or interfere with their operation. In addition, mylar sheets are easy to handle and can be cut, shaped, and formed to fit the specific requirements of the application. In summary, mylar sheets are used to provide insulation for various components in a circuit because of their high dielectric strength, tensile strength, and resistance to moisture, chemicals, and UV radiation. They act as a barrier that separates the components from each other and from the external environment, helping to prevent thermal stress, degradation of performance, and interference with other components in the circuit. The versatility and durability of mylar sheets make them an ideal choice for a wide range of applications in the electronics industry.

## Proposed prototype watch circuit

**Figure 13:**
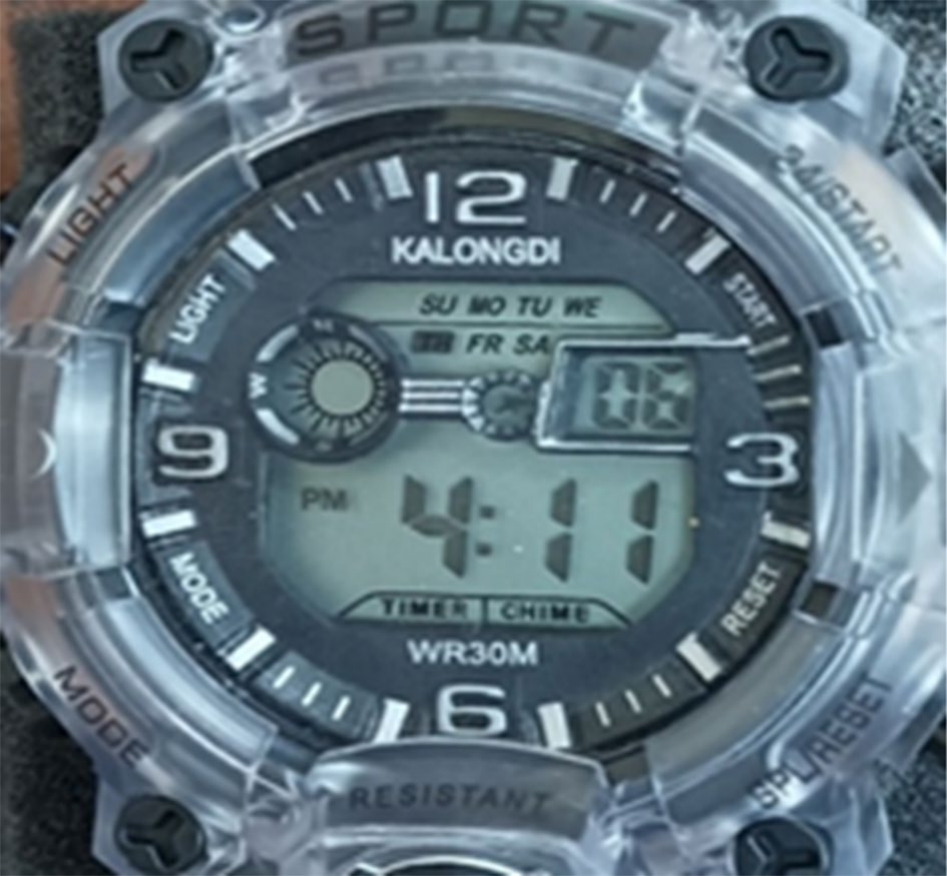
Digital Watch.

**Figure 14:**
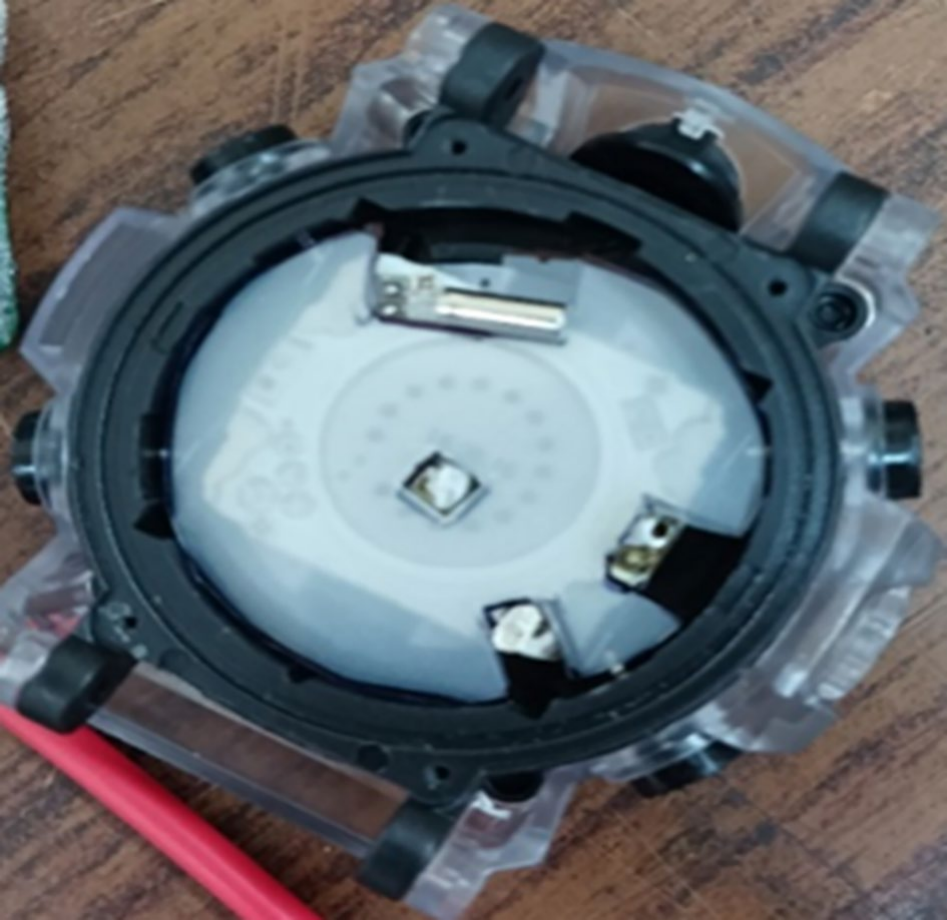
Terminals of the Watch Circuit.

The work uses a digital watch that displays time in 12-h or 24-h format, as well as seconds, date, day, and alarm indicators, and a blinking design that changes every second. This watch also features a stop watch and allows users to set the time, date, and alarms as can be seen in Fig. 10. Additionally, it has a multi-colored LED. Upon acquiring the watch, the battery was removed, and the battery terminals were inspected as seen in Fig. 13. Subsequently, the rechargeable battery's dropped voltage terminals were connected to the appropriate terminals on the watch, providing a suitable power source for the circuit. To ensure ease of wearability, the placement of all components in the watch is crucial. The step-up circuit and the battery circuit are positioned underneath the watch circuit. The TEG is situated on the opposite side of the wrist where the watch is typically worn. To bring all these elements together, a single wide rubber strap/band, as depicted in Fig. 14 is utilized.

**Figure 15:**
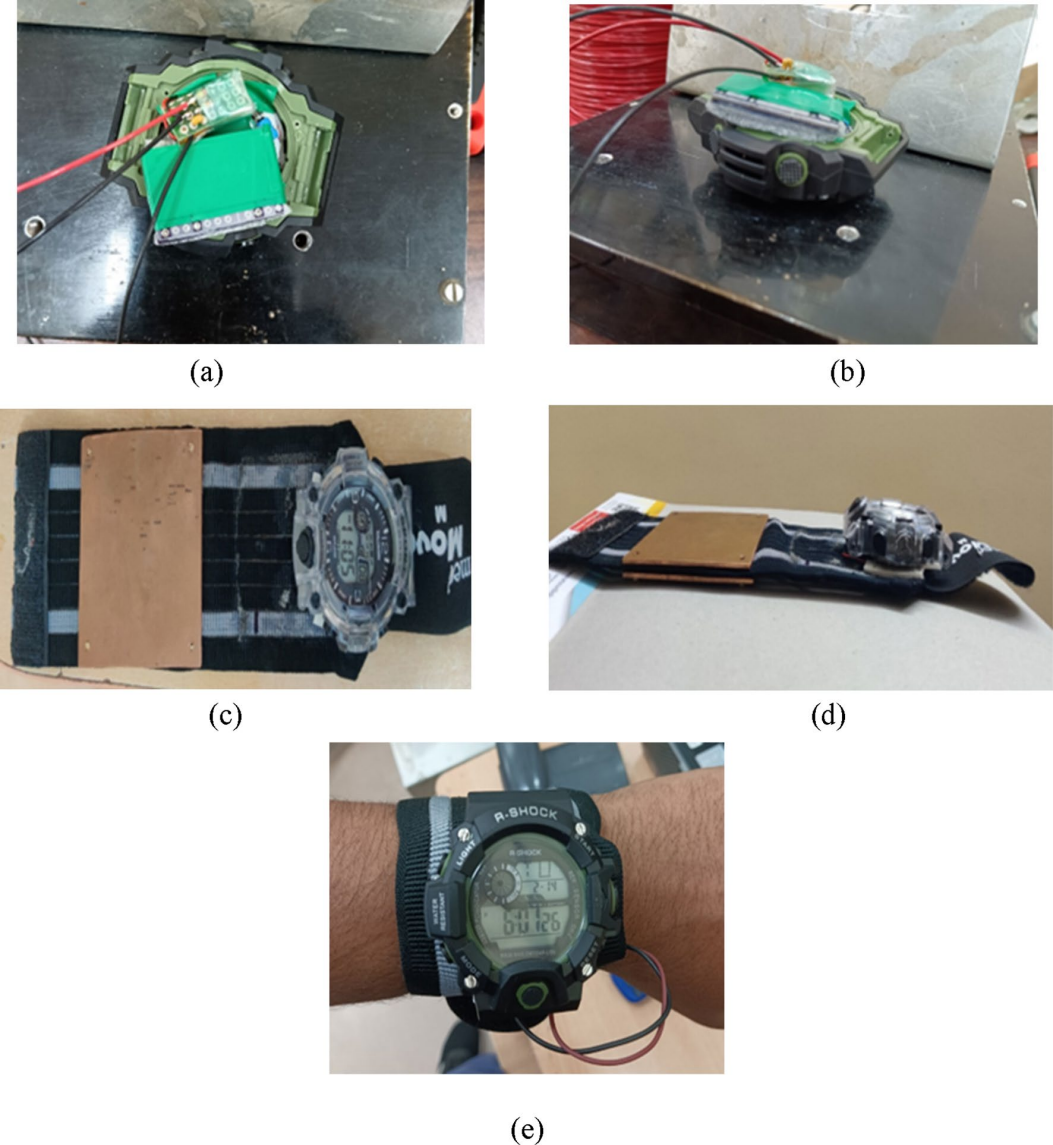
Different stages of prototype development. (**a**) Fixing TEG, (**b**) Stepup circuit and battery circuit, (**c**) Fixing copper plate-top view, (**d**) Copper plate front view, (**e**) Complete timepiece setup.

The prototype arrangement depicted in Fig. 15a–e guarantees that the watch is not only functional but also comfortable to wear. Assembling all the components of the digital watch prototype was done successfully, determining the most effective way to create a circuit and insulate the components to ensure optimal functionality.

The step-up circuit and battery circuit were then positioned under the watch circuit, resulting in a thickness of 1 mm greater than traditional watches. To mitigate any potential bulkiness, the copper plates are incorporated with the TEG on the opposite side into the belt, resulting in an additional thickness of only 6 mm. Although the watch may be wider than conventional models due to the inclusion of this specific TEG, the benefits of utilizing lost heat energy to generate voltage are noteworthy. This technology could significantly reduce greenhouse gas emissions and contribute to the development of more sustainable energy solutions.

## Experimental results and discussions

**Figure 16:**
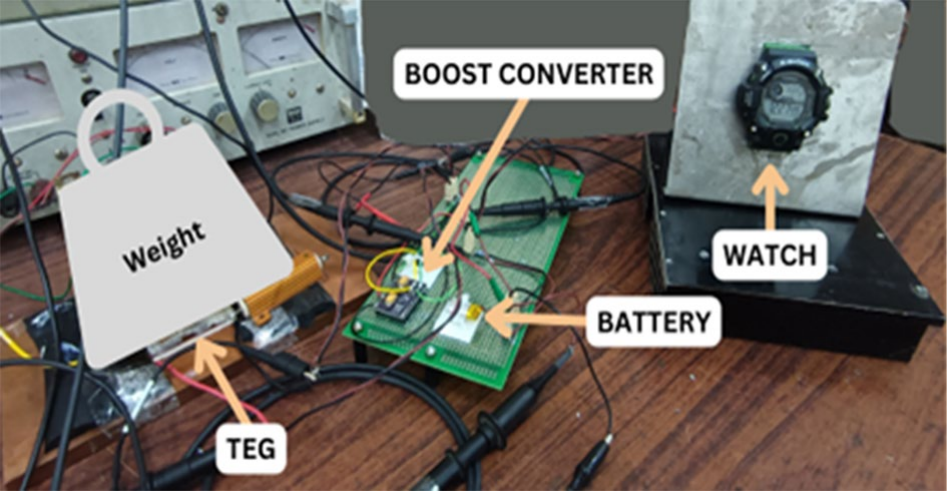
Experimental Setup of the proposed prototype.

Upon completion of component assembly, the watch functions properly, incorporating vital features including a stopwatch, special stop, alarm clock, as well as time, date, and day settings. Figure 16 illustrates the experimental setup established to measure the voltage readings at crucial components of the circuit. The comprehensive assessment of the performance of the TEG by utilizing a high-precision multimeter is conducted. This allowed us to obtain accurate readings for the temperature on both sides of the TEG, as well as the voltage generated by it.

Additionally, the voltage converted by the boost converter was measured and Table 1 gives details of the voltage generated from TEG for different values of T_c_ and T_h_.

**Table 1:** Voltage generated by TEG.

Tc (°C)	Th (°C)	Va (mV)	Vb (V)
25.6	27.5	100	3.16
27	31	120	3.20

**Figure 17:**
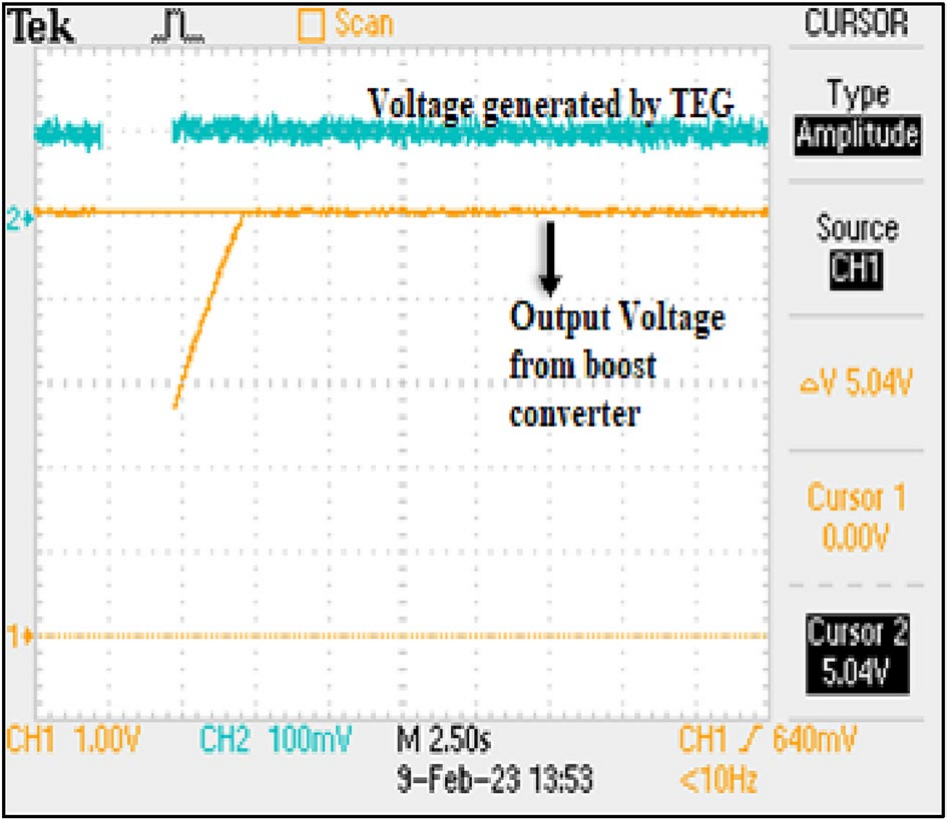
Oscilloscope output at Tc = 25.0 °C and Th = 27.2 °C.

**Figure 18:**
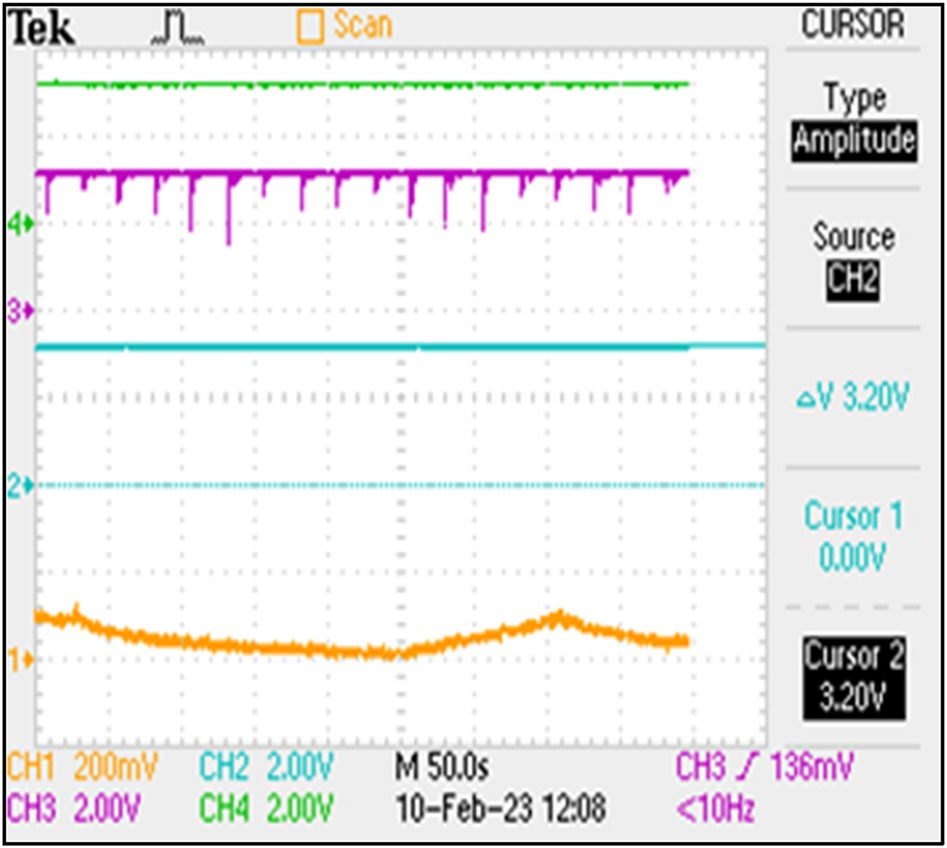
Oscilloscope graph-2.

In Table 2—Tc = 25.0 °C and Th = 27.2 °C and the corresponding values are observed from DSO. Based on the data presented in CH1, it is observable from the graphical representation that the TEG exhibits at least a minimum voltage output of 90 mV, while the temperature differential ranges from 1 to 3 degrees. In CH2, it is evident that upon connection of the TEG, the voltage converted by the boost converter exhibits an initial rise from 2.96 to 5.04 V within a short period of 2.5 s, subsequently demonstrating a stable and regulated voltage output. Based on the observations made in CH1, it can be inferred that the TEG is generating a voltage within the range of 16–112 mV, depending on the temperature differential exhibited between its two sides. Figure 17 and 18 shows the oscilloscope output and graph, respectively.

**Table 2:** Oscilloscope Reading-1.

Time (s)	0	2.5	5	7.5	10	12.5	15	17.5	20
CH1 (V)	2.96	5.04	5	5.08	5.04	5.04	5.04	5.04	5.04
CH2 (V)	0.105	0.106	0.104	0.104	0.104	0.102	0.102	0.102	0.102

**Figure 19:**
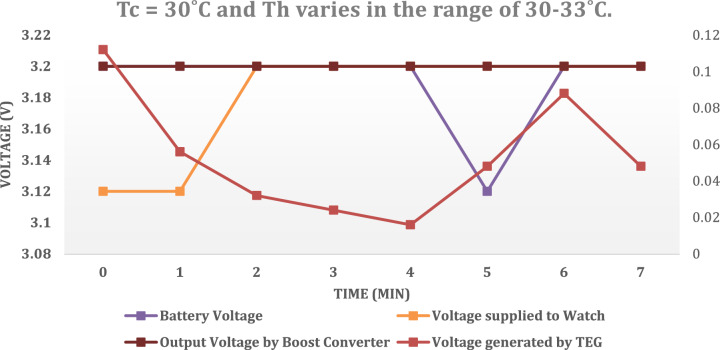
Performance of TEG watch at Tc = 30 °C and Th varies in the range of 30–33 °C.

This temperature differential ranges from 0 to 3 °C, thus indicating a direct correlation between the temperature gradient and voltage output. Regarding CH2, it has been observed that the battery voltage is gradually increasing from 3.12 to 3.2 V, indicating a steady charge over time. Furthermore, CH3 and CH4 exhibit the same behaviour as CH2, given that the battery, boost converter, and voltage supply to the watch are interconnected and functionally dependent on one another. In Fig. 19, Tc remains constant at 30 °C and Th varies in the range of 30–33 °C.

In this case (Table 3), Tc remains constant at 30 °C and Th varies in the range of 30–33 °C. Based on these subsequent readings taken approximately two hours later, it can be confirmed that the battery is indeed undergoing a charging process, as evidenced by an increase in voltage from 3.12 to 3.28 V. The maximum output power obtained and analysed is around 11.5 W to 14.5 W. The variation in the power output is due change in internal resistance, load resistance and differential temperature between hot and cold end.The thermoelectric conversion efficacy of the proposed TEG module is found to be 5.0–5.7%.

**Table 3:** Experimental results.

Time (mins)	0	1	2	3	4	5
Voltage generated by TEG (V)	0.092	0.104	0.112	0.104	0.088	0.104
Battery voltage (V)	3.2	3.2	3.2	3.28	3.2	3.2
Voltage supplied to watch (V)	3.2	3.2	3.2	3.2	3.2	3.2
Output voltage by boost converter (V)	3.28	3.28	3.2	3.2	3.2	3.28

For Hot end temperature at 40 °C and cold end temperature at 35 °C (ΔT = 5 °C), Table 4 shows the analysis of output power obtained for varaible load resistance.

**Table 4:** Experimental results—Power generated at Hot end temperature 40 °C and cold end temperature 35 °C.

Load resistance (Ω)	Output voltage (V)	Output current (A)	Output power (W)
2	5.1	2.2	11.22
2.5	4.6	2.0	9.2
3	3.8	1.27	4.82
3.5	3.6	1.03	3.71
4	3.4	0.85	2.89

The Table 4 shows the estimated output voltage, current, and power at different load resistances for a ΔT of 5 °C. As the load resistance increases, the output current decreases. The maximum output power is achieved when the load resistance matches the internal resistance of the TEG.

Table 5 displays the comparison of TEG-19913 with other commercial TEGs interms of maximum ΔT, Output Voltage (V), Output Current (A), Output Power (W) and Temperature Range (°C).

**Table 5:** Comparison with other commercial TEGs.

Parameter	TEG1-19913	TXL-199-02Q	TEG2-126LDT
Maximum ΔT (°C)	≥ 63	70	85
Maximum Output Voltage (V)	23.4 (estimated)	16.2	12.6
Maximum Output Current (A)	13	15	10
Maximum Output Power (W)	11.5	15.8	10.5
Operating Temperature Range (°C)	− 50 ~ 80	− 40 ~ 120	− 40 ~ 120
Size (mm)	40 × 40 × 3.8	40 × 40 × 3.0	40 × 40 × 3.0

From Table 5 it is observed that the TEG1-19913 offers a higher maximum ΔT compared to TXL-199-02Q and TEG2-126LDT, potentially leading to higher theoretical output power under suitable conditions. However, TEG1-19913's estimated maximum output voltage is lower than TXL-199-02Q. TEG2-126LDT has the lowest output current and power among the three.

## Conclusion and future work

Thus, a digital watch is developed that employs the heat energy emanating from the human body to power its functionality. This is achieved using a TEG that generates voltage by leveraging the temperature differential across its surfaces, which is amplified using a boost converter and utilized to charge a 3.7 V rechargeable battery, which serves as the primary power source for the watch circuit. The maximum output power obtained is around 11.5W to 14.5W and the thermoelectric conversion efficacy is found to be 5.0—5.7%. The watch performs satisfactorily, with features including stopwatch, alarm clock, time and date, and day setting operating properly. The successful demonstration of TEGs as a useful tool in harnessing lost heat energy to generate voltage represents a critical step towards building a sustainable and environmentally-friendly future. This proof-of-concept represents a valuable contribution to the field of renewable energy and showcases the potential for further advancements in this area. Although the TEG uses the temperature differential across its surfaces to generate voltage from the heat energy emitted by the human body, it can sometimes generate a negative potential when ambient temperature surpasses body heat in some tropical locations. Addressing this issue is critical for future prototypes to ensure optimal performance. Additionally, methods for amplifying current delivery at the designated voltage should be incorporated as well, to resolve the issue of the LED light not illuminating. Furthermore, the current model's weight is attributed to the use of copper plates, a heavy metal. These challenges must be effectively addressed to engineer a more advanced and efficient version of this watch.

### Supplementary Information

Supplementary Information.

## Data Availability

All data generated or analysed during this study are included in this main article (and it is alos submitted as Supplementary Information files).
