# Supplementary material for: Thermoelectric generator powered timepiece circuit for rechargeable battery operation
Source: Sci Rep. 2024 Apr 15;14:8668. doi: 10.1038/s41598-024-59260-8 (PMC11018777; doi:10.1038/s41598-024-59260-8)
Supplement: Supplementary file 1 — Supplementary Information. [file 41598_2024_59260_MOESM1_ESM.docx]

EXPERIMENTAL SETUP

Upon completion of component assembly, the watch functions properly, incorporating vital features including a stopwatch, special stop, alarm clock, as well as time, date, and day settings.

MULTIMETER READINGS

To begin with, we conducted a comprehensive assessment of the performance of the TEG by utilizing a high-precision multimeter. This allowed us to obtain accurate readings for the temperature on both sides of the TEG, as well as the voltage generated by it. Additionally, we also measured the voltage that was converted by the boost converter. We have compiled a table detailing the readings we recorded during our assessment. The following table provides a clear and concise representation of the data we collected.

| **Tc (°C)** | **Th (°C)** | **Va (mV)** | **Vb (V)** |
| --- | --- | --- | --- |
| 25.6 | 27.5 | 100 | 3.16 |
| 27 | 31 | 120 | 3.20 |

*Table 1. Multimeter Readings*

Note- The oscilloscope readings displayed on the graph starts at the second horizontal division from the left-hand side and extends all the way to the right end of the graph. Once it reaches the end, the waveform circles back to the first and the last reading is near the end of the first horizontal division.

In this case- Tc= 25.0˚C and Th= 27.2˚C

CH1: Output Voltage by Boost Converter

CH2: Voltage generated by Thermoelectric Generator

| **Time (s)** | **0** | **2.5** | **5** | **7.5** | **10** | **12.5** | **15** | **17.5** | **20** |
| --- | --- | --- | --- | --- | --- | --- | --- | --- | --- |
| **CH1 (V)** | 2.96 | 5.04 | 5 | 5.08 | 5.04 | 5.04 | 5.04 | 5.04 | 5.04 |
| **CH2 (V)** | 0.105 | 0.106 | 0.104 | 0.104 | 0.104 | 0.102 | 0.102 | 0.102 | 0.102 |

*Table 2. Oscilloscope Reading-1*

Based on the data presented in CH1, it is observable from the graphical representation that the thermoelectric generator (TEG) exhibits at least a minimum voltage output of 90mV, while the temperature differential ranges from 1 to 3 degrees. In CH2, it is evident that upon connection of the TEG, the voltage converted by the boost converter exhibits an initial rise from 2.96V to 5.04V within a short period of 2.5 seconds, subsequently demonstrating a stable and regulated voltage output.

In this case, Tc remains constant at 30˚C and Th varies in the range of 30-33˚C.

CH1: Voltage generated by Thermoelectric Generator

CH2: Battery Voltage

CH3: Voltage supplied to Watch

CH4: Output Voltage by Boost Converter

| **Time (mins)** | **0** | **1** | **2** | **3** | **4** | **5** | **6** | **7** |
| --- | --- | --- | --- | --- | --- | --- | --- | --- |
| **CH1 (V)** | 0.112 | 0.056 | 0.032 | 0.024 | 0.016 | 0.048 | 0.088 | 0.048 |
| **CH2 (V)** | 3.2 | 3.2 | 3.2 | 3.2 | 3.2 | 3.12 | 3.2 | 3.2 |
| **CH3 (V)** | 3.12 | 3.12 | 3.2 | 3.2 | 3.2 | 3.2 | 3.2 | 3.2 |
| **CH4 (V)** | 3.2 | 3.2 | 3.2 | 3.2 | 3.2 | 3.2 | 3.2 | 3.2 |

*Table 3. Oscilloscope Reading-2*

In this case, Tc remains constant at 30˚C and Th varies in the range of 30-33˚C.

CH1: Voltage generated by Thermoelectric Generator

CH2: Battery Voltage

CH3: Voltage supplied to Watch

CH4: Output Voltage by Boost Converter

Based on these subsequent readings taken approximately two hours later, it can be confirmed that the battery is indeed undergoing a charging process, as evidenced by an increase in voltage from 3.12V to 3.28V.

After performing comprehensive tests and conducting thorough analyses, we have substantiated that the anticipated outcomes from our experimental setup have been achieved. We can confidently assert that our proposed system operates effectively.

| **Time (mins)** | **0** | **1** | **2** | **3** | **4** | **5** |
| --- | --- | --- | --- | --- | --- | --- |
| **CH1 (V)** | 0.092 | 0.104 | 0.112 | 0.104 | 0.088 | 0.104 |
| **CH2 (V)** | 3.2 | 3.2 | 3.2 | 3.28 | 3.2 | 3.2 |
| **CH3 (V)** | 3.2 | 3.2 | 3.2 | 3.2 | 3.2 | 3.2 |
| **CH4 (V)** | 3.28 | 3.28 | 3.2 | 3.2 | 3.2 | 3.28 |

*Table 4. Oscilloscope Reading-3*
